# Genomic and Phenotypic Characterization of Avian-Derived *Limosilactobacillus reuteri* Strains Showing Pathogen-Inhibiting Activity and Folate Production

**DOI:** 10.3390/ani16132039

**Published:** 2026-07-02

**Authors:** Taís Mayumi Kuniyoshi, Iago Blanco, João Victor dos Anjos Almeida, Carlos Emilio Cabrera Matajira, Ana Clara Candelaria Cucick, Taciana Freire de Oliveira, Sabrina da Silva Sabo, Elionio Galvão Frota, Pamela Oliveira de Souza de Azevedo, Fernando Moises Mamani Sanca, Marcos Camargo Knirsch, Mauro de Medeiros Oliveira, Alessandro de Mello Varani, Ricardo Pinheiro de Souza Oliveira

**Affiliations:** 1 Department of Biochemical-Pharmaceutical Technology, School of Pharmaceutical Sciences, University of São Paulo (USP), Rua Do Lago, 250, Cidade Universitária, São Paulo 05508-000, SP, Brazil; t.kuniyoshi@alumni.usp.br (T.M.K.); iagoblanco@usp.br (I.B.); k.rlos89.cabrera@gmail.com (C.E.C.M.); anaclara.candelaria@usp.br (A.C.C.C.); taciana.oliveira@usp.br (T.F.d.O.); sabrinasabo@gmail.com (S.d.S.S.); egalvaofrota@gmail.com (E.G.F.); pam.o.souza@gmail.com (P.O.d.S.d.A.); fernando.mamani.ms@gmail.com (F.M.M.S.); marcos.knirsch@usp.br (M.C.K.); 2 Department of Agricultural and Environmental Biotechnology, College of Agricultural and Veterinary Sciences, São Paulo State University (UNESP), Jaboticabal 14884-900, SP, Brazil; joao.anjos@unesp.br (J.V.d.A.A.); mauromedeiros@alumni.usp.br (M.d.M.O.); alessandro.varani@unesp.br (A.d.M.V.)

**Keywords:** lactic acid bacteria, pathogen-inhibiting agents, prebiotic, folate

## Abstract

**Simple Summary:**

Antibiotic resistance is a growing concern in animal production, increasing the need for safer alternatives. This study evaluated two beneficial bacterial strains isolated from the gut of broiler chickens. Both strains inhibited selected harmful bacteria, produced folate, and grew in the presence of prebiotic fibers, suggesting potential for future feed formulations. Safety tests showed no harmful traits, such as the ability to destroy blood cells or known disease-causing genes. However, they were found to carry a tetracycline resistance gene, which must be removed or controlled before practical use. Overall, the study identified promising chicken-derived bacterial strains while reinforcing the importance of genomic safety screening in probiotic development.

**Abstract:**

The escalating global concern regarding bacterial antibiotic resistance in animal production has intensified the search for sustainable and effective alternatives to conventional antimicrobials. In this study, two *L. reuteri* strains (LBM-Ti195 and LBM-Ti173) are isolated from broiler cecal microbiota that were characterized through an integrated approach, combining phenotypic assays with comparative genomic analysis. Both strains exhibited antibacterial activity against relevant veterinary and foodborne pathogens, including *Listeria monocytogenes* CECT 934, *Staphylococcus aureus* CECT 239, *Clostridium perfringens* Type A, and *Campylobacter jejuni* CCAMP 162. The inhibitory activity anti-*S. aureus* increased by more than 10% modifying cultivation conditions, while comparative genomic analysis identified an M23-family metallopeptidase as a potential candidate for pathogen inhibition. Phenotypically, both strains produced folate and metabolized fructooligosaccharides (FOS) and inulin, supporting their potential compatibility with synbiotic formulations. Genome reconstruction reinforces these functional findings by revealing a complete predicted de novo folate biosynthesis pathway. In addition, CAZyme annotation identified higher copy numbers of glycosyltransferases GT2 and GT4 compared with the reference strains, suggesting differences in cell-surface carbohydrate metabolism and exopolysaccharide (EPS)-associated traits. Safety profiling revealed no hemolytic activity or conserved virulence factors under the tested conditions. However, phenotypic tetracycline resistance was detected, and in silico analysis identified an acquired *tetW* gene in a putative plasmid-associated context, highlighting the importance of an in-depth evaluation of strains with probiotic potential. Collectively, these findings position LBM-Ti195 and LBM-Ti173 as promising avian-derived *L. reuteri* candidates for next-generation zootechnical probiotic development, while highlighting antimicrobial resistance (AMR) mitigation and further functional validation as essential steps toward safe application.

## 1. Introduction

The identification of strategies to address the escalating emergence of antibiotic-resistant bacterial lineages constitutes an imperative scientific and public health priority. This endeavor is contextualized within the One Health [1] paradigm, which acknowledges the intrinsic interconnectedness of human, animal, and environmental health systems. Given that approximately 70% of globally commercialized antimicrobial agents are allocated to animal production systems, the restructuring of agrifood production chains represents a pivotal intervention strategy to mitigate the dissemination of AMR [2].

Consequently, the search for alternatives to antibiotic use in animal production, without compromising productivity and food safety, has been one of the major challenges faced by the scientific community over recent decades. The use of probiotics has become established in the market as a recurrent practice to promote animal health and support zootechnical performance [3,4,5]. Their mechanisms of action include competitive exclusion of pathogens, strengthening of mucosal barrier integrity, and immunomodulation [5,6]. Many of the strains currently commercialized as probiotics belong to the lactic acid bacteria (LAB), a functionally defined rather than taxonomically uniform group of Gram-positive bacteria naturally present in numerous fermented foods of plant, meat, and dairy origin, as well as in the host intestinal microbiota, where they may establish host-associated interactions [6].

A classic example of host-associated diversification is *L. reuteri* (recently reclassified from *Lactobacillus reuteri* [7,8]), a gut-associated symbiont whose evolutionary history is characterized by genomic diversification and lineage specialization associated with specific vertebrate hosts [8,9]. This adaptation may contribute to intestinal persistence through cell-surface proteins and EPS, which can participate in mucosal adhesion, biofilm formation, and competitive interactions with other microorganisms [10]. *L. reuteri* further stands out for its fermentative capacity to generate strain-dependent pathogen-inhibiting agents—particularly proteins/peptides (e.g., bacteriocins) and secondary metabolites—that inhibit clinically relevant veterinary and zoonotic pathogens, such as *Escherichia coli*, *L. monocytogenes*, *S. aureus*, and *C. perfringens* [11,12,13].

Numerous studies have highlighted the health-promoting properties of *L. reuteri*, with several strains established as widely used probiotics. The literature reports benefits including improved intestinal health [14] and modulation of the gut–brain–microbiota axis [15]. In poultry, dietary supplementation with *L. reuteri* and other LAB probiotic strains [16,17,18] has emerged as a promising alternative to antibiotic growth promoters. *L. reuteri* strain SL001 has been associated with improved growth performance, immune function, oxidative stress responses, intestinal morphology, and gut microbial composition. However, these effects are strongly strain-dependent and cannot be generalized to all isolates within the species [18].

In addition to these probiotic-associated properties, certain *L. reuteri* strains exhibit the metabolic autonomy to synthesize folate (vitamin B9) de novo from simple precursors via their own enzymatic machinery, independent of external dietary sources. Microbially produced folate may contribute to host nutrition by acting at the intestinal mucosa, where it serves as an essential cofactor for DNA synthesis, cell division, and tissue development. Given the high cellular turnover of the intestinal epithelium, local folate availability could potentially support enterocyte proliferation, intestinal integrity, and mucosal homeostasis [19].

The contemporary intensive poultry production system is generally characterized by high bird density, a factor that can significantly increase the risk of zoonotic pathogen dissemination and, consequently, the necessity for antimicrobial agents. Therefore, the present study conducted a bioprospecting screening of two LAB strains isolated from the intestinal microbiota of broiler chickens (*Gallus gallus domesticus*). The selection of host-derived strains was prioritized to increase the likelihood of isolating candidates capable of surviving gastrointestinal transit and exhibiting adhesion to the intestinal mucosa within the specific avian gut environment. These candidate strains were selected based on their pathogen-inhibitory activity against Gram-positive and Gram-negative bacteria of veterinary and zoonotic significance, alongside their metabolic ability to synthesize folate de novo. Furthermore, recognizing the requirement for rigorous safety assessment of zootechnical additives, this investigation performed a phenotypic and genomic characterization of the isolates to evaluate their probiotic-associated traits, biosafety profile, and potential for future zootechnical application.

## 2. Materials and Methods

### 2.1. In Vitro Assays

#### 2.1.1. Sampling of Chicken Cecum and LAB Isolation and Initial Characterization

For the isolation of LAB, ten healthy 12-week-old broiler chickens (*Gallus gallus domesticus*) were selected. The inclusion criteria required birds from the same commercial flock, showing normal development, and maintained on a commercial feed containing animal protein, free of antibiotic growth promoters (AGP-free). Their diet consisted of commercial feeds formulated according to the nutritional recommendations commonly used in the Brazilian poultry industry for the different growth phases. These diets were based primarily on corn and soybean meal as the main sources of energy and protein, respectively, and were supplemented with vegetable oils, minerals, vitamins, and amino acids to meet the birds’ nutritional requirements [20]. Feed and water were provided ad libitum [21] throughout the rearing period. Diet formulation and nutrient levels were consistent with the recommendations described in the Brazilian Tables for Poultry and Swine [20]. Birds that had experienced any infections or had been treated with antibiotics were excluded from the study. Animals were euthanized via an anesthetic overdose (barbiturate) under approval of the Ethics Committee on the Use of Animals of the USP (registration number 7843030717). Following euthanasia, the ceca were aseptically harvested, and all ceca were completely homogenized to obtain a uniform pool of cecal contents. A 10 g aliquot of this homogenized pool was then diluted in 90 mL of sterile peptone water and homogenized using a Stomacher. Serial 10-fold dilutions were prepared and plated on de Man, Rogosa and Sharpe (MRS) agar supplemented with 0.1 g/L of cycloheximide and incubated anaerobically using the GasPak™ EZ Anaerobe Container System with indicator (BD Diagnostics, Sparks, MD, USA) at 37 °C for 48 h.

For the preliminary selection of LAB, colonies initially isolated from the MRS plates were subjected to three biochemical assays, as previously described by Sabo et al. [21], to confirm their presumptive LAB identity. To perform the catalase test, a small amount of colony growth was mixed with one drop of 3% hydrogen peroxide (Sigma-Aldrich^®^, Merck KGaA, Darmstadt, Germany) on a microscope slide, with the absence of bubble formation indicating a negative result for enzyme catalase presence. For gelatinase activity, each isolate was cultivated in a gelatin medium containing 3% (*w*/*v*) gelatin, 0.5% (*w*/*v*) peptone, 0.3% (*w*/*v*) yeast extract, and 0.3% (*w*/*v*) beef extract, incubated at 37 °C for 24 h, and subsequently cooled at 4 °C for 1 h; the absence of liquefaction indicated a negative result. Finally, coagulase production was evaluated by mixing 500 μL of overnight culture with 500 μL of rabbit plasma (COAGU-PLASMA, Laborclin, Pinhais, Brazil) and incubating at 37 °C for 24 h, using *S. aureus* CECT 239 as a positive control, where the absence of plasma coagulation (solidification) confirmed a negative reaction as previously described by de Souza de Azevedo et al. [22]. All assays were performed in triplicate.

#### 2.1.2. Hemolytic Activity

To evaluate hemolytic activity, overnight cultures of the selected isolates were streaked onto Blood Agar plates supplemented with 5% sheep blood (Difco, BD, Franklin Lakes, NJ, USA) and incubated at 37 °C for 48 to 72 h under microaerophilic conditions [22]. Hemolysis was classified based on the appearance of clear zones around the colonies indicating β-hemolysis (complete lysis), greenish zones indicating α-hemolysis (partial lysis), or no visible change indicating γ-hemolysis (non-hemolytic reaction). *S. aureus* CECT 239 was used as the reference control strain for β-hemolysis [22].

#### 2.1.3. In Vitro Acid and Bile Salt Tolerance

As a preliminary screening, in vitro survival under simulated gastrointestinal conditions was assessed by subjecting the isolates to acidic and bile salt challenges in modified MRS broth, according to Frota et al. [23] with minor modifications. Specifically, the MRS broth was adjusted to pH 2.0 for the acid tolerance challenge and supplemented with 0.3% (*w*/*v*) bile salts (Sigma-Aldrich, St. Louis, MO, USA) for the bile tolerance assay. Samples from both assays were incubated at 37 °C and, after 2 h of exposure, serially diluted in decimal steps (10-fold) using sterile saline solution (0.85% *w*/*v* NaCl) as the diluent, and subsequently plated on MRS agar to determine cell viability. Survival rates, expressed in colony-forming units per milliliter (CFU/mL), were compared to a control group incubated under the same conditions in unmodified MRS broth (pH 6.0 and 0% bile salts). An isolate was considered tolerant if its cell count remained within the same logarithmic unit as the control group.

#### 2.1.4. Identification Using Mass Spectrometry Identification (MALDI-TOF MS)

Isolates were preliminarily identified by Matrix-Assisted Laser Desorption/Ionization–Time of Flight Mass Spectrometry (MALDI-TOF MS) using the Biotyper^®^ system (Bruker Daltonics, Bremen, Germany), following the manufacturer’s instructions. Briefly, bacterial colonies were transferred to a steel target plate, treated with 1 μL of 70% formic acid, and overlaid with 1 μL of α-cyano-4-hydroxycinnamic acid (CHCA) matrix. Calibration was performed using *E. coli* DH5α as the bacterial test standard. Mass spectra were acquired in linear positive ion mode using an N_2_ cartridge laser at 60 Hz. Species-level identification was assigned based on log(score) values ≥ 2.0 against the Biotyper^®^ database. Final taxonomic confirmation was subsequently supported by whole-genome-based analyses.

#### 2.1.5. Antimicrobial Susceptibility Profiling

To assess antimicrobial susceptibility, the Minimum Inhibitory Concentration (MIC) of nine clinically relevant antibiotics—ampicillin, clindamycin, chloramphenicol, erythromycin, gentamicin, kanamycin, streptomycin, tetracycline, and vancomycin (Inlab Diagnóstica, Alomax Ltda, São Paulo, Brazil)—was determined via the broth microdilution method in triplicate, following the European Food Safety Authority (EFSA) guidelines [24]. Inocula were prepared to obtain a final density 10^5^ CFU/mL in LSM medium (9:1 Cation-Adjusted Mueller-Hinton Broth and MRS broth), according to Klare et al. [25]. Microtiter plates were incubated at 37 °C for 24 h. The isolates were categorized as resistant or susceptible using the species-specific microbiological cut-off values (breakpoints) established in the latest EFSA guidance on the characterization of microorganisms [24]. Quality control was ensured using *E. coli* ATCC 25922 and *S. aureus* ATCC 29213 as reference strains, in compliance with Clinical and Laboratory Standards Institute (CLSI) standards [26].

#### 2.1.6. Evaluation of Pathogen-Inhibitory Activity of Cell-Free Supernatants Against Indicator Microorganisms

The pathogen-inhibitory activity of the produced cell-free supernatants (CFS) was evaluated against the following indicator microorganisms: *L. monocytogenes* CECT 934, *S. aureus* CECT 239, *C. jejuni* CCAMP 162, *S.* Heidelberg IOC 969/17, *Escherichia coli* ATCC 25922, and *C. perfringens* A-IB/CPDIV/Cp-A/s 01-07 (Type A).

To obtain the CFS, each *L. reuteri* strain was cultivated in MRS broth at 37 °C for 24 h under anaerobic conditions. The bacterial cultures were subsequently centrifuged at 4470× *g* for 10 min at 4 °C. To stabilize the organic acids produced during lactic fermentation, the pH of the recovered CFS was adjusted to 6.0–6.5 using 1 M sodium hydroxide (NaOH). Finally, the supernatant was exposed to 70 °C for 3 min to achieve complete enzyme and thermolabile peptide inactivation [27]. To determine the proteinaceous nature of the inhibitory substances present, cell-free supernatant (CFS) samples exhibiting pathogen-inhibitory activity were treated with the proteolytic enzymes trypsin (2 mg/mL) at 37 °C for 2 h, followed by a thermal inactivation step at 70 °C for 20 min. The remaining activity was evaluated against bioindicators, with those samples completely losing their inhibitory capacity after enzymatic treatment being classified as harboring proteinaceous pathogen-inhibitory agent.

Pathogen-inhibitory activity was determined based on the percentage of inhibition according to a methodology adapted from Linares-Morales et al. [28] Initially, the inoculum concentration of each indicator microorganism was standardized by optical density at 600 nm, corresponding to approximately 10^6^ CFU/mL. Subsequently, 100 μL of the produced CFS of *L. reuteri* LBM-Ti173 or LBM-Ti195 was added to 100 μL of the standardized inoculum in sterile 96-well microplates. The plates were incubated at 37 °C for 18 h.

For each assay, a negative control consisting of MRS without the addition of pathogenic microorganisms was included. The positive control consisted only of the culture medium and the inoculum growth medium. All assays were performed in triplicate. The percentage of inhibition was calculated according to Equation (1):

$$\%inhibition=(\left[OD\right]b/\left[OD\right]a)\times 100$$

where:

$$\left[OD\right]a=\left[OD\right]positive\;control-\left[OD\right]negative\;control$$

$$\left[OD\right]b=\left[OD\right]sample-\left[OD\right]negative\;control$$

#### 2.1.7. Factorial Screening of Culture Conditions Affecting Pathogen-Inhibitory Activity

To preliminarily evaluate the influence of environmental culture conditions on the pathogen-inhibitory activity of the isolates, a two-level full factorial design (2^3^) was performed. Three variables were assessed: incubation temperature (37 °C and 42 °C), culture medium pH (5.0 and 6.5), and agitation rate (80 and 200 rpm). The experimental combinations were distributed into eight assays (C1–C8) using coded low (−1) and high (+1) levels for each factor as follows: C1 (+1, +1, −1), C2 (+1, −1, −1), C3 (+1, −1, +1), C4 (−1, +1, −1), C5 (−1, +1, +1), C6 (−1, −1, +1), C7 (+1, +1, +1), and C8 (−1, −1, −1), corresponding to temperature, pH, and agitation, respectively.

The inoculum concentration was standardized by optical density at OD_600_, corresponding to approximately 10^6^ CFU/mL, and cultures were incubated for 18 h under the respective experimental conditions. After cultivation, cell-free supernatants were obtained and their inhibitory activity was evaluated against *S. aureus* CECT 239 through growth inhibition assays.

Pathogen-inhibitory activity was determined based on the area under the bacterial growth curve (AUC), and the inhibition percentage was calculated by comparing the reduction in AUC of each treatment relative to the positive growth control:

$$\%inhibition=(\left[AUC\right]control-\left[AUC\right]treatment)/\left[AUC\right]control\;\times 100$$

To evaluate the contribution of each experimental variable to pathogen-inhibitory activity, factor effects were estimated as the difference between the mean inhibition percentage obtained at the high level (+1) and the mean inhibition percentage obtained at the low level (−1) for each factor according to:

$$Effect\_i=\left[Y’\right]high-\left[Y’\right]low$$

where $\left[Y’\right]high\;$ and $\left[Y’\right]low$ correspond to the mean inhibition percentages obtained under high and low levels of each experimental factor, respectively.

#### 2.1.8. Evaluation of Folate (Vitamin B9) Production

The ability of the bacterial strains to produce folate was initially screened in folate-free medium (FACM) according to the method described by Cucick et al. [29] and Pacheco da Silva et al. [30], with minor modifications. Briefly, strains were first activated in their respective growth media at 37 °C for 24 h, harvested by centrifugation (10,000× *g*, 5 min), washed three times with sterile saline solution, and resuspended to their original culture volume. Subsequently, aliquots (4% *v*/*v*) were inoculated into 3 mL of FACM (HiMedia Laboratories, Mumbai, India) and incubated at 37 °C for 24 h. This adaptation procedure was repeated for seven consecutive passages. Strain growth was monitored visually by optical density after each passage, and strains showing growth throughout all seven passages were considered presumptive folate producers.

Following the presumptive screening, samples from positive strains were collected after the seventh passage and 24 h of incubation for the determination of extracellular (EC) and intracellular (IC) folate concentrations, as previously described by Laiño et al. [31,32]. For EC folate extraction, culture supernatants were mixed (1:1 v/v) with protective buffer containing 0.82% sodium acetate and 1% ascorbic acid. For IC folate extraction, cell pellets were washed, resuspended in the same protective buffer, and both EC and IC samples were heat-treated (100 °C for 5 min), centrifuged, and stored at −20 °C until analysis.

Folate concentrations were determined by microbiological assay using the folate-dependent and chloramphenicol-resistant strain *Lacticaseibacillus rhamnosus* NCIB 10463 as the indicator microorganism, according to Cucick et al. [33] and Laiño et al. [31]. The indicator strain was sequentially adapted to FACM supplemented with chloramphenicol and used as inoculum for the assay. Samples were diluted in 0.1 M phosphate buffer (pH 7.0) according to a folic acid standard curve (0–1.0 μg/L), transferred in triplicate to 96-well microplates, and mixed with an equal volume of the indicator suspension. After incubation at 37 °C for 48 h, microbial growth was measured at 600 nm using a microplate reader, and folate concentrations were calculated from the standard curve and expressed as [ng/mL].

#### 2.1.9. Evaluation of Different Prebiotic Concentrations on Bacterial Growth

Bacterial growth was evaluated in glucose-free MRS medium. Three different prebiotics were tested as alternative carbon sources: fructooligosaccharides (FOS), mannanoligosaccharides (MOS), and inulin. Each prebiotic was evaluated at concentrations of 1%, 2%, and 3% (*w*/*v*).

The assays were carried out in sterile 96-well microplates. Wells containing MRS medium supplemented with the respective prebiotic concentrations were inoculated with the bacterial culture. MRS medium without glucose and without prebiotic supplementation was used as the negative control. Microplates were incubated at 37 °C without agitation, and bacterial growth was monitored for 20 h using a microplate reader. Optical density was measured at 600 nm (*OD*: 600 nm) at regular intervals (30 min) throughout the incubation period. All treatments were performed in triplicate.

#### 2.1.10. Statistical Analysis

Statistical analyses were applied to phenotypic and in vitro assays. Experiments were performed in triplicate, and the results were expressed as mean ± standard deviation (SD). Statistical differences between treatments were evaluated using one-way analysis of variance (ANOVA). Whenever a significant primary effect was detected, Tukey’s post-hoc test was subsequently applied for multiple comparisons, with statistical significance accepted at p≤0.05. All analyses were conducted using Statistica 8.0 software (StatSoft Inc., Tulsa, OK, USA).

### 2.2. Genome Analysis

#### 2.2.1. Genome Sequencing, Processing and Assembly

Genomic DNA from isolates LBM-Ti173 and LBM-Ti195 was extracted using the Wizard^®^ Genomic DNA Purification kit (Promega, Madison, WI, USA) and quantified using a Qubit fluorimeter (Life Technologies, Carlsbad, CA, USA). Paired-end libraries were prepared using the Nextera XT DNA Library Preparation kit (Illumina, San Diego, CA, USA) and sequenced on the Illumina MiSeq platform using the MiSeq Reagent v3 kit (600 cycles).

The quality of the raw reads was assessed using FastQC v0.11.9. Subsequently, the reads were subjected to fastp v0.20.1 [34] for adapter removal and quality filtering, using Phred ≥30 and a minimum length of 36 bp. De novo assembly was performed using Unicycler v0.5.1 [35,36,37]). Contigs with a length of less than 1000 bp were discarded using seqtk v1.3 [36].

#### 2.2.2. Plasmid Assembly and Identification

Putative plasmid-associated sequences were investigated using plasmid-targeted assembly with the plasmidSPAdes mode implemented in the St. Petersburg Genome Assembler (SPAdes) v4.0.0 [38,39], using the quality-filtered Illumina reads generated for each isolate. The resulting contigs were screened against the PlasmidFinder database v2.1 using ABRicate v1.4.0 to identify known plasmid replicon sequences. In parallel, contigs were analyzed with PLASMe v1.1 [40] a machine learning-based tool for plasmid sequence identification and classification. Contigs harboring antimicrobial resistance genes were further inspected for plasmid-associated features, including predicted replication genes, mobility-related genes, sequencing coverage, and genomic context. Because plasmid circularization was not experimentally confirmed, plasmid assignments were interpreted as putative.

#### 2.2.3. Quality Evaluation and Functional Annotation of Genomic Data

The quality of the assemblies was assessed with QUality ASsessment Tool (QUAST) v5.3.0 [41], considering contiguity metrics, including number of contigs, total genome size, N50, and GC%. Completeness was estimated with Benchmarking Universal Single-Copy Orthologs (BUSCO) v6.0.0 [42], using the lactobacillales_odb10 database, and completeness/contamination was also checked with CheckM v1.2.4 [43].

Structural annotation was performed using the NCBI Prokaryotic Genome Annotation Pipeline (PGAP) [44], which produced standardized annotation files and coding DNA sequences (CDS) predictions for subsequent analyses. For comparative functional annotation and reconstruction of metabolic pathways, the genomes were subjected to BlastKOALA v3.1 [45] and Rapid Annotations using Subsystems Technology (RAST) [46], which assigns gene functions within SEED subsystems (v2.0) and produces an initial reconstruction of central and specialized metabolism. Gene classification by orthologous groups was performed with eggNOG-mapper v2.

#### 2.2.4. Average Nucleotide Identity (ANI)

Pairwise ANI values were previously computed including LBM-Ti173, LBM-Ti195, and 73 complete RefSeq reference genomes, using FastANI v1.33 under default parameters (fragment length: 3000 bp; minimum fraction of bidirectional mappings: 0.2) [47]. The resulting similarity matrix was imported and post-processed in Python v3 using the pandas library v2.2 [48]. Species-level identity was assessed using the accepted ANI ≥ 95% threshold relative to the type strain *L. reuteri* DSM 17938 (GCF_041888805.1), which served as the species reference [49]. The complete matrix was visualized as a hierarchical clustermap using the seaborn library, with Unweighted Pair Group Method with Arithmetic Mean (UPGMA) hierarchical clustering applied according to pairwise ANI similarity [50]. The final figure was further refined in Inkscape v1.4.4.

#### 2.2.5. In Silico Safety Assessment of Strains’ Genomes

AMR genes were screened with several gene mining tools, including ResFinder v4.7.2 [51], ABRicate v1.4.0 [52], and Resistance Gene Identifier (RGI v6.0.5) based on Comprehensive Antibiotic Resistance Database (CARD v4.0.1) [53], which were applied under standard parameters, inferring the predicted phenotypes based on detected genes. Hits with high identity and coverage were considered, and the results of the three tools were compared in an integrated manner.

Prophage elements were screened for in query genomes using the PHASTEST tool v3.0 [54] under standard parameters, generating lists of phage genes, alignment results, and phage region maps. Additionally, virulence-associated genes were identified by alignment against the Virulence Factor Database v5.0 (VFDB), enabling detection of virulence factors (VFs) and their functional categories [55]. Finally, the pathogenic potential towards humans was estimated with PathogenFinder v2 [56], which applies machine-learning models to sequences and reports the probability of human pathogenicity.

A protocol adapted from the public pipeline “FAMR” (Frequency of Acquired Antimicrobial Resistance Genes) [49] was applied to characterize the abundance and distribution of sequences in all complete genomes and chromosomes of *L. reuteri* annotated within RefSeq. Sequences identified in strains were used as input. tblastn (BLAST 2.14.1+) was performed against this database of genomes, with a cutoff E-value of 1 × 10^−5^ [57]. Hits with identity and coverage ≥80% of the reference sequence were considered significant.

#### 2.2.6. Gene Mining and Prediction of Biosynthetic Gene Clusters (BGC)

The genetic prospection of biosynthetic gene clusters (BGCs) potentially associated with the production of secondary metabolites was performed using antiSMASH v6 [58]. The detection criterion was set to strict, and all extra features were enabled, including ClusterBlast, SubClusterBlast, KnownClusterBlast, and specific domain detection. Results were inspected in the antibiotics & Secondary Metabolite Analysis Shell (antiSMASH) graphical interface, and predicted BGCs were annotated according to cluster type, genomic range, and predicted gene architecture.

The prediction of loci associated with bacteriocins and other ribosomally synthesized and post-translationally modified peptides (RiPPs) was performed using the BAGEL4 pipeline [59]. Because automated bacteriocin annotations may be affected by incomplete database representation and low-confidence matches, predicted bacteriocin-related proteins were further manually inspected using BLASTP analysis [57].

#### 2.2.7. Functional Reconstruction of Metabolic Pathways

Functional reconstruction of metabolic capabilities was performed by combining Kyoto Encyclopedia of Genes and Genomes (KEGG) and SEED subsystem-based annotations. Protein sequences were first annotated with evolutionary genealogy of genes: Non-supervised Orthologous Groups (eggNOG)-mapper v2 [60] to obtain orthology assignments, Clusters of Orthologous Groups (COG) categories, and KEGG Orthology (KO) identifiers, and then submitted to BlastKOALA for pathway mapping within the KEGG framework [45]. In parallel, genomes were explored in the SEED Viewer v2.0 [46] to group genes into curated subsystems. SEED feature subsystems were analyzed based on the presence or absence of genes across genomes to infer pathway completeness and potential metabolic functions. Biosynthetic pathways for folate (vitamin B9) were reconstructed based on annotations obtained with the NCBI PGAP [44] and further refined.

Enzyme assignments were mapped onto KEGG reference metabolic pathways to guide pathway inference [45]. Computational support for selected enzyme annotations was obtained through two complementary approaches: (i) domain identification with InterProScan v5.75-106.0 [61] and (ii) structural modeling with AlphaFold 3 [62], following preprocessing with SignalP 6.0 [63] when signal peptide prediction was required. Model quality was assessed using the SAVES v6.1 PROCHECK tool [64], and structural inspections, comparisons, and alignments were carried out with PyMOL v3.1.3 [65] and FATCAT v2 [66], using manually curated UniProt reference sequences as benchmarks [67].

#### 2.2.8. Genomic Context Analysis

The genomic organization of ARGs, BGCs and vitamin biosynthetic operons was manually examined using Locus Visualisation tool (LoVis4u) v0.1.4.1 [68], enabling functional contextualization of the gene clusters. For each locus, genomic regions comprising 20 kb upstream and downstream of the target operon or gene were extracted from the annotated assemblies whenever permitted by the contiguity of the genome. These regions were analyzed in comparison with probiotic strains with demonstrated activity in chickens: *L. reuteri* strain ATCC PTA-126788 (GCF_020978285.1) and ATCC PTA-126787 (GCF_020978225.1); pigs: *L. reuteri* strain I5007 (GCF_000410995.1); and humans: *L. reuteri* strain ATCC PTA 5289 (GCF_040962025.1); and *L. reuteri* strain CRL 1098 (GCF_001657495.1) in food. Additionally, strains capable of biosynthesizing vitamins B2 (AMBV339, GCF_940926095.1), B9 (JCM 1112, GCF_000010005.1), and B12 (NCDC958, GCF_046763525.1) were included for comparative purposes.

All analyses were performed with default parameters unless otherwise specified. Genomic context comparisons were interpreted as computational evidence for locus conservation, disruption, or association with mobile genetic elements, and not as experimental confirmation of gene function or metabolic activity.

#### 2.2.9. Carbohydrate-Active Enzymes (CAZymes) Predicted Terms

The repertoires of CAZymes were characterized with DataBase for automated Carbohydrate-active enzyme ANnotation (dbCAN3) v4.1.4 [69], combining results from the profile Hidden Markov Models (HMMER) [70], dbCAN-sub [69], and DIAMOND [71] modules. To improve accuracy, only annotations supported by at least two of these three methods were considered for downstream analyses.

#### 2.2.10. Data Availability and Accession Numbers

The genome assembly of *L. reuteri* LBM-Ti173 and *L. reuteri* LBM-Ti195 and associated raw data have been deposited in the National Center for Biotechnology Information (NCBI) GenBank database under the BioProject accession number PRJNA1472526.

## 3. Results

### 3.1. Bioprospecting and Genome Identification

#### 3.1.1. Preliminary In Vitro Bioprospecting

Morphological and biochemical characterization was performed on 314 isolates obtained from broiler chicken cecal contents using selective MRS medium. The screening process followed a rigorous stepwise elimination: initially, all 314 isolates were screened for pathogen-inhibitory activity against *S. aureus* CECT 239, resulting in the selection of 34 pathogen-inhibiting candidates. To confirm the nature of the inhibitory compounds, cell-free supernatants were treated with proteinase K, which neutralized the activity in 15 isolates, identifying them as producers of proteinaceous antimicrobial agents. These 15 isolates were further evaluated for safety and biochemical markers, including Gram-positive staining, absence of hemolytic activity, and negative reactions for catalase, coagulase, and gelatinase, narrowing the selection to 10 non-hemolytic LAB isolates. Finally, these candidates were screened for vitamin B9 production, leading to the selection of only two target strains, LBM-Ti173 and LBM-Ti195, which were the only isolates to produce folate (Table 1). Both strains were subsequently identified as *L. reuteri* by MALDI-TOF MS [log(score) ≥ 2.0] and characterized as heterofermentative, producing lactic and acetic acids. Furthermore, both isolates demonstrated phenotypic tolerance to simulated gastrointestinal conditions, maintaining high viability after exposure to acidic pH and bile salts (Table 1).

#### 3.1.2. LBM-Ti173 and LBM-Ti195 Genomic Identification

- Genome assembling and quality

Sequencing quality inspection showed that both strains presented Phred base quality scores predominantly above 30 across the read length. After quality filtering, LBM-Ti195 retained 5,314,768 reads (Q30: 94.88%), while LBM-Ti173 retained 5,490,208 reads (Q30: 95.09%). LBM-Ti195 assembled into 75 contigs, with a total length of 2,093,541 bp. LBM-Ti173 assembled into 163 contigs, spanning 2,061,835 bp. Assemblies returned 99.8% BUSCO completeness and 99.46% CheckM completeness with 0.14% contamination (Table 2).

Structural annotation predicted 2103 CDSs, 20 tRNAs, 2 rRNAs, 4 ncRNAs, 1 tmRNA, 17 pseudogenes and 2 sORFs for LBM-Ti195, with a coding density of 88.9%. For LBM-Ti173, annotation yielded 2,085 CDSs, 17 tRNAs, 2 rRNAs, 4 ncRNAs, 1 tmRNA, 17 pseudogenes and 2 sORFs, with a coding density of 89.3%. Complete genome assembly and annotation metrics are summarized in Table 2.

- Taxonomic identification and comparative genomic analysis

**Figure 1:**
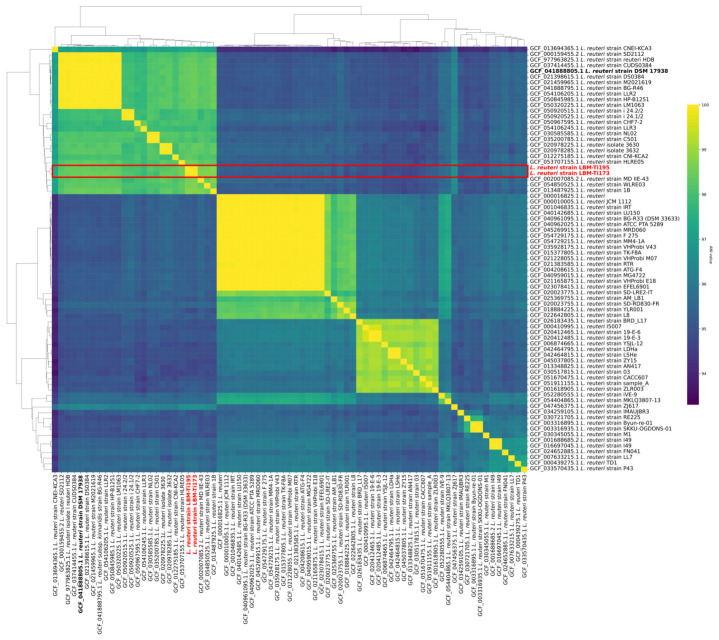
Average Nucleotide Identity (ANI) clustermap of *L. reuteri* genomes retrieved from NCBI RefSeq. Pairwise ANI values (%) are represented by a color gradient ranging from dark purple, indicating lower similarity values (~94%), to yellow, indicating higher similarity values (100%), as shown in the scale bar. Hierarchical clustering was performed using the UPGMA method based on pairwise ANI distances and is intended to summarize genomic similarity among the analyzed strains. The genome of *L. reuteri* DSM 17938, used as the species-level reference type strain is indicated in bold. The two *L. reuteri* strains sequenced in the present study (LBM-Ti195 and LBM-Ti173) are highlighted in red text and indicated by the red box.

ANI comparisons of LBM-Ti195 and LBM-Ti173 against 73 complete *L. reuteri* RefSeq genomes supported species-level assignment for both isolates, with pairwise ANI values ranging from 94.66% to 98.80%. The two isolates shared a mutual ANI of 99.99%, indicating near-identical genomic backgrounds. Compared with the probiotic reference strain *L. reuteri* strain DSM 17938 (GCF_041888805.1), ANI values were 98.41% and 98.44% for LBM-Ti195 and LBM-Ti173, respectively. The closest reference genome for both isolates was *L. reuteri* 1B (GCF_013487925.1), with ANI values of 98.80% and 98.73%, respectively. Overall, 22 and 21 reference genomes shared ≥98% ANI with LBM-Ti195 and LBM-Ti173, respectively, reflecting close intraspecific relatedness within a genomically diverse species. The hierarchical clustermap derived from the all-against-all ANI matrix revealed genomic subclusters within *L. reuteri* (Figure 1). Both LBM-Ti195 and LBM-Ti173 co-clustered within the same genomic subgroup of reference strains and were positioned near DSM 17938.

### 3.2. Safety Profile

#### 3.2.1. Antimicrobial Susceptibility Profile of L. reuteri LBM-Ti173 and LBM-Ti195

The phenotypic antimicrobial susceptibility profile of *L. reuteri* LBM-Ti173 and LBM-Ti195 was determined by MIC assays, following EFSA technical requirements for microorganisms intended for use as zootechnical additives (Table 3). Both strains were classified as susceptible (S) to most of the clinically relevant antibiotics tested, with MIC values below the established microbiological cut-off values for ampicillin, gentamicin, kanamycin, streptomycin, clindamycin, chloramphenicol, and erythromycin. However, both isolates exhibited tetracycline MIC values above the established cut-off value, indicating phenotypic resistance and requiring genomic investigation to determine whether this resistance was intrinsic or associated with acquired antimicrobial resistance determinants. Vancomycin resistance was also observed in both strains; however, this phenotype is widely recognized as intrinsic in *Limosilactobacillus* spp., and EFSA does not establish cut-off values for this antibiotic.

#### 3.2.2. In Silico Antimicrobial Resistance Assessment and Pathogenic Genes

Following an EFSA-oriented AMR screening workflow, *tetW* was identified as the only antimicrobial resistance determinant consistently detected in both LBM-Ti195 and LBM-Ti173 across the analytical tools used [49]. CARD-RGI identified *tetW* (ARO:3000194) with 96.71% predicted identity, while ABRicate/ResFinder detected the same determinant with 98.96% sequence identity. This gene encodes a tetracycline resistance ribosomal protection protein associated with resistance to tetracycline and related compounds, including doxycycline, minocycline, chlortetracycline, demeclocycline, and oxytetracycline.

To evaluate the distribution of *tetW* within *L. reuteri*, a species-level comparative screening was performed using RefSeq genomes sharing >95% ANI with the reference genome set used in this study. After ANI-based filtering, 67 genomes were retained for analysis. Within this dataset, *tetW* was detected in 11 genomes from diverse host sources and geographic origins (Table S1). The limited and uneven distribution of *tetW* among species-level *L. reuteri* genomes supports its classification as an acquired AMR determinant. Regarding genomic location, eight *tetW*-positive genomes were found to carry the gene in chromosomal contexts (GCF_053797155.1, GCF_04745675.1, GCF_022642805.1, GCF_0200978225.1, GCF_051670475.1, and GCF_001618905.1), whereas three carried it in plasmid-associated contexts after BLAST analysis [57]. This variable genomic localization further supports the association of *tetW* with mobile or horizontally acquired elements.

In the isolates analyzed in this study, the fragmented nature of the draft assemblies initially prevented precise determination of whether *tetW* was integrated into the chromosome or associated with plasmid sequences. To further investigate this issue, plasmid-targeted assembly was performed using plasmidSPAdes [38], which recovered a 17,595 bp contig harboring *tetW* in both LBM-Ti195 and LBM-Ti173. Although PlasmidFinder did not detect known plasmid replication genes from its database, both contigs exhibited relatively high sequencing coverage, consistent with a possible multicopy plasmid-associated sequence. In addition, classification with PLASMe [40], a machine learning-based plasmid prediction tool, yielded high plasmid probability scores of approximately 0.99 for both contigs.

**Figure 2:**
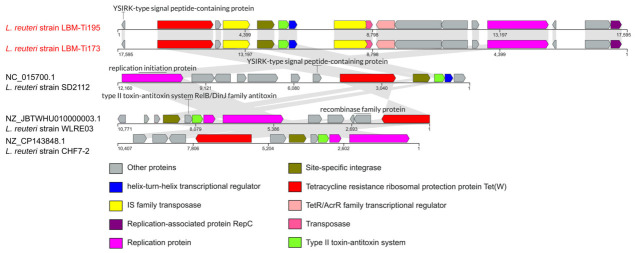
Genomic context of the tetracycline resistance gene *tetW* in *L. reuteri* LBM-Ti173 and LBM-Ti195 are highlighted in red and compared with plasmid-associated regions. The comparison supports a putative plasmid-associated context for *tetW* in the two isolates.

This prediction was further supported by synteny and genomic context analyses. In Figure 2, the *tetW* insertion context in LBM-Ti195 and LBM-Ti173 was associated not only with mobility-related elements, including transposases and recombinases, but also with plasmid-associated replication genes, including replication protein and RepC. Furthermore, synteny analysis of three plasmid-associated RefSeq sequences revealed the presence of replication protein genes, resembling the context observed in the two isolates (Figure 2). Collectively, these results support a putative plasmid-associated location for *tetW* in both LBM-Ti195 and LBM-Ti173.

In contrast, putative vancomycin resistance-associated hits showed less than 40% identity and were therefore not considered relevant AMR determinants under the applied screening criteria. This interpretation is also consistent with the intrinsic vancomycin resistance phenotype commonly described in *Limosilactobacillus* spp. and with the absence of EFSA cut-off values for this antibiotic.

For virulence-associated gene screening, no conserved virulence factors were detected in either genome under the applied identity and coverage thresholds. In addition, PathogenFinder classified both isolates as non-pathogenic to humans. These results support the absence of major known virulence-associated determinants in the analyzed genomes, although they do not replace experimental safety assessment.

### 3.3. Probiotic-Associated Traits of L. reuteri LBM-Ti173 and LBM-Ti195

#### 3.3.1. Inhibitory Spectrum of Cell-Free Supernatants

- Inhibitory Spectrum of CFS from *L. reuteri* LBM-Ti173 and LBM-Ti195

**Figure 3:**
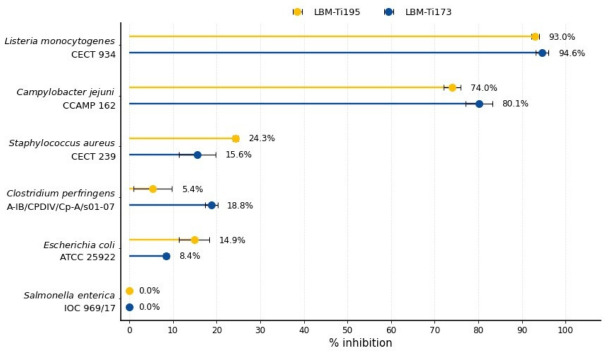
Inhibition profile (%) of cell-free supernatants (CFS) produced by *L. reuteri* LBM-Ti173 (blue) and LBM-Ti195 (yellow) against bacterial indicators relevant to zootechnical production and food safety.

The pathogen-inhibitory activity of the CFS produced by both strains LBM-Ti173 and LBM-Ti195 was evaluated against indicator bacteria associated with animal production and food safety (Figure 3). Both CFS exhibited strong inhibitory activity against *L. monocytogenes* CECT934, with inhibition values of 94.6 ± 1.5% and 93.0 ± 0.9% for LBM-Ti173 and LBM-Ti195, respectively. Among the Gram-negative bacteria, Campylobacteriosis-associated *C. jejuni* CCAMP162 showed substantial susceptibility, with inhibition rates of 80.1 ± 3.1% for LBM-Ti173 and 74.0 ± 2.0% for LBM-Ti195. Moderate inhibition was observed against *S. aureus* CECT239 (15.6 ± 4.2% and 24.3 ± 0.7%) and *C. perfringens* A-IB/CPDIV/Cp-A/s01-07 (18.8 ± 1.4% and 5.4 ± 4.4%) for LBM-Ti173 and LBM-Ti195, respectively. In contrast, *Escherichia coli* ATCC 25922 showed low susceptibility to both CFS, with inhibition values of 8.4 ± 0.7% and 14.9 ± 3.5%, respectively. No inhibitory activity was detected against *Salmonella* enterica subsp. enterica strain IOC969/17.

- Pathogen-Inhibitory Activity Under Different Culture Conditions

The full factorial design allowed the evaluation of variations in pathogen-inhibitory activity associated with changes in pH, temperature, and agitation against *S. aureus* CECT 239. As a descriptive complement to the experimental design, factor effects were estimated as the difference between the mean inhibition percentage obtained at the high (+1) and low (−1) levels of each factor. For isolate LBM-Ti173, the calculated effects were +4.11 for temperature, −16.81 for pH, and −5.53 for agitation. For isolate LBM-Ti195, the calculated effects were +10.46 for temperature, −11.39 for pH, and −6.96 for agitation. These values indicate that, within the tested experimental range, pH had the strongest effect on inhibitory activity for both isolates, with higher inhibition observed under the lower pH condition.

**Figure 4:**
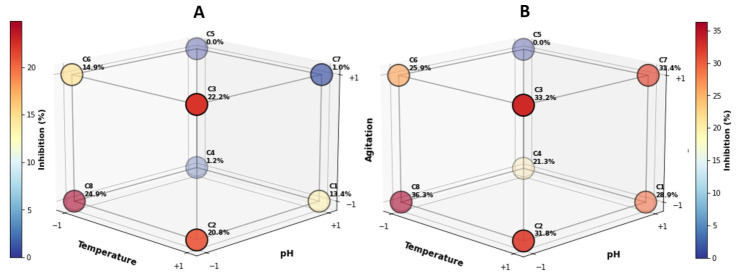
Three-dimensional factorial cube representation of the influence of culture conditions on the pathogen-inhibitory activity of isolates LBM-Ti173 (**A**) and LBM-Ti195 (**B**) against *S. aureus* CECT239. Conditions C1–C8 represent the experimental combinations evaluated using coded factor levels (+1 and −1) for temperature (42 and 37 °C), pH (6.5 and 5.0), and agitation rate (200 and 80 rpm), respectively. The color scale represents the inhibition percentage (%) calculated from the area under the growth curve (AUC), with warmer colors (red/orange) indicating higher inhibition and cooler colors (blue) indicating lower inhibition.

The inhibition percentages obtained under the different experimental combinations are presented in Figure 4 and in the corresponding factorial matrices. In the three-dimensional factorial cube representations, each point corresponds to a specific experimental condition (C1–C8), defined by the combination of coded levels for temperature, pH, and agitation. The spatial position of each point reflects only the experimental combination evaluated, whereas the associated color represents the inhibition percentage calculated from the AUC for that condition.

For isolate LBM-Ti173 (Figure 4A), the highest inhibitory activity was observed under condition C8 (24.86 ± 2.31%), followed by C3 (22.23 ± 1.84%); both conditions differed significantly from the positive growth control (*p* < 0.05). Similarly, isolate LBM-Ti195 (Figure 4B) showed the highest inhibitory activity under condition C8 (36.31 ± 3.12%), which differed significantly from the positive growth control (*p* < 0.05). Although condition C3 also presented a high inhibition percentage (33.21 ± 2.47%), it did not reach statistical significance compared with the positive growth control (*p* = 0.091). Thus, C8, corresponding to 37 °C, pH 5.0, and 80 rpm, represented the most favorable condition among those tested, but should not be interpreted as a fully optimized production condition.

Additionally, treatment of the CFS preparations with trypsin resulted in complete loss of inhibitory activity, supporting the involvement of proteinaceous pathogen-inhibitory agents in the observed inhibition.

- Gene mining of potentially bioactive regions

Genome screening with BAGEL4 predicted two areas of interest (AOIs) in each strain, initially annotated as Enterolysin A variant 63.3 and Enterolysin A variant 64.3. However, the predicted core peptides showed low-confidence matches, with identity values ranging from 36.9% to 39.0% (Table S2).

Manual BLASTP inspection showed that the predicted core peptide within the Enterolysin A variant 63.3 AOI aligned to a *L. reuteri* phage tail protein with 96.58% sequence identity.

Further PHASTEST analysis predicted intact prophage regions colocalized with this AOI in both strains, with a PHASTEST score of 100 and similar prediction metrics. LBM-Ti195 was predicted to harbor two intact prophage regions, whereas LBM-Ti173 was predicted to harbor one (Table S3). These findings suggest that the Enterolysin_A variant 63.3 AOI is more likely associated with prophage-related genes than with a canonical bacteriocin locus.

In contrast, the predicted core peptide within the Enterolysin_A variant 64.3 AOI aligned to a *L. reuteri* M23-family metallopeptidase with 98.36% identity. Given that M23-family metallopeptidases may participate in peptidoglycan hydrolysis, this protein represents a plausible candidate associated with the proteinaceous pathogen-inhibitory activity observed in the CFS assays. However, its direct contribution to this activity remains to be experimentally demonstrated.

Additionally, antiSMASH analysis predicted one Type III polyketide synthase (T3PKS) biosynthetic gene cluster in each strain. Both clusters showed identical hits for the core biosynthetic gene. The antiSMASH annotation classified this gene as containing a Chal_sti_synt_N domain, corresponding to the N-terminal chalcone/stilbene synthase domain, with manual BLASTP curation indicating similarity to hydroxymethylglutaryl-CoA synthase at 93.58% identity in both strains. Because the pathogen-inhibitory activity was abolished after trypsin treatment, this T3PKS-like cluster is unlikely to explain the observed proteinaceous inhibition under the tested conditions.

#### 3.3.2. Folate Production

- Intracellular and extracellular vitamin B9 production

Both strains showed growth throughout the seven consecutive passages in folate-free medium (FACM), indicating presumptive folate production. Quantification of extracellular (EC), intracellular (IC), and total folate further supported the ability of both strains to synthesize folate under these conditions (Table 4).

Despite the strain-dependent nature of folate production, both strains showed similar extracellular, intracellular, and total folate concentrations under the tested conditions, with only slight variations. Total folate production ranged from 42.1 ± 4.6 to 50.8 ± 4.7 ng mL^−1^. The strain *L. reuteri* LBM-Ti195 showed the highest total folate concentration (50.8 ± 4.7 ng mL^−1^), as well as higher extracellular (36.8 ± 5.2 ng mL^−1^) and intracellular (14.1 ± 0.5 ng mL^−1^) folate concentrations compared with *L. reuteri* LBM-Ti173. For both strains, extracellular folate represented the major fraction of the total folate produced (Table 4).

- In silico reconstruction of B9-vitamin biosynthetic pathways

Genomic reconstruction of the folate biosynthesis pathway identified 13 KO entries corresponding to relevant genes, with identical profiles in LBM-Ti195 and LBM-Ti173 (Table S2). Metabolic entries were distributed across functionally related modules: (i) the de novo folate biosynthesis pathway and (ii) the molybdopterin cofactor (MoCo) biosynthesis pathway, both of which are connected to GTP-derived metabolic intermediates.

**Figure 5:**
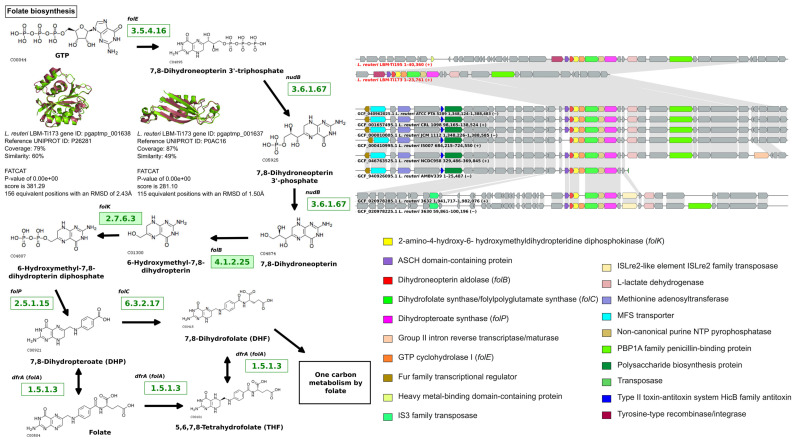
Reconstruction of the vitamin B9 biosynthesis pathway in *L. reuteri* LBM-Ti195 and LBM-Ti173. The metabolic map displays the predicted intermediates and enzymes involved in the pathway. Structural models generated for selected enzymes are shown in green, while reference structures used for comparison are shown in magenta, with alignment and overlap statistics indicated. The right panel shows the genomic context of the corresponding biosynthetic genes, highlighting conserved synteny among *L. reuteri* reference strains and the sequenced strains LBM-Ti195 and LBM-Ti173.

Six genes associated with folate biosynthesis were identified in both genomes: *folA*, *folB*, *folC*, *folE*, *folK*, and *folP*. The *folE*, *folB*, *folK*, and *folP* genes are involved in the biosynthesis of pterin-derived intermediates and dihydropteroate, whereas *folC* and *folA* participate in folate polyglutamylation and terminal reduction steps, respectively. In addition, a Nudix hydrolase gene (*nudB*) was identified, which may contribute to the conversion of 7,8-dihydroneopterin triphosphate to 7,8-dihydroneopterin [72]. Together, the presence of these genes supports the predicted capacity for de novo synthesis of tetrahydrofolate-polyglutamate derivatives in both strains. The presence of two *folC*-like paralogs in each genome is consistent with the functional distinction previously described in *L. reuteri* between FolC-associated folate polyglutamylation and FolC2-associated synthesis of 5,10-methenyltetrahydrofolate polyglutamate (5,10-CH=THF). Finally, *folA* encodes dihydrofolate reductase, which reduces DHF to tetrahydrofolate (THF), a biologically active folate derivative that serves as a substrate for one-carbon metabolism (Figure 5).

The conservation of flanking *loci* around the *folE–folB–folK–folC–folP* cluster, observed in comparison with *L. reuteri* reference strains in the right panel of Figure 5, supports the conservation of this genomic organization in the studied strains and in reference isolates recognized as folate producers. Additionally, the bifunctional gene *ribBA* (K14652; EC:4.1.99.12/3.5.4.25), mapped in KEGG map00790 as encoding GTP cyclohydrolase II, was identified as the only representative of the riboflavin pathway in this reconstruction. Six genes associated with the molybdopterin cofactor biosynthesis pathway (*moaA*, *moaB*, *moaC*, *moaE*, *moeA*, and *mobA*), also mapped in KEGG map00790 and linked to GTP-derived metabolism, were identified in both genomes with conserved *loci* (Table S2).

In addition to the de novo folate biosynthesis pathway, 11 genes associated with the “one carbon pool by folate” module (KEGG map00670) were identified in both strains with identical profiles (Table S2). These genes included *folA* (EC:1.5.1.3), involved in DHF/THF interconversion; *folD* (EC:1.5.1.5/3.5.4.9); *glyA/SHMT* (EC:2.1.2.1); *thyA/TYMS* (EC:2.1.1.45); *purH* (EC:2.1.2.3/3.5.4.10); *purN* (EC:2.1.2.2); *metK/MAT* (EC:2.5.1.6); *mmuM/BHMT2* (EC:2.1.1.10); *fhs* (EC:6.3.4.3); *MTHFS* (EC:6.3.3.2); and *lpd/pdhD* (EC:1.8.1.4) (Table S2). The simultaneous identification of genes associated with KEGG map00790 and map00670 supports the predicted connection between de novo THF biosynthesis and one-carbon metabolism in both strains, including pathways related to purine, thymidylate, methionine, and S-adenosylmethionine metabolism (Figure 5).

#### 3.3.3. Prebiotic Fermentation

- Growth of *L. reuteri* LBM-Ti195 and LBM-Ti173 on FOS, MOS, and Inulin as Carbon Source

**Figure 6:**
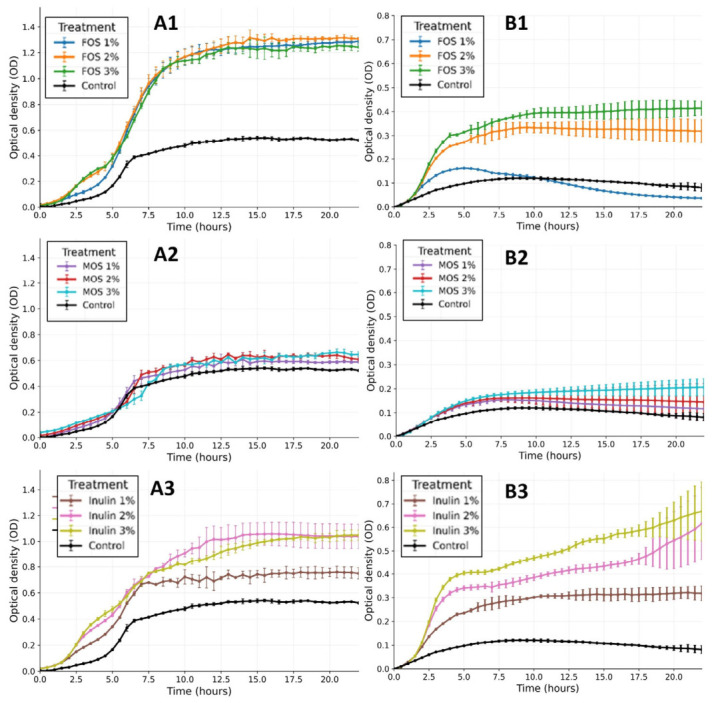
Growth curves of isolates LBM-Ti173 and LBM-Ti195 cultivated in the presence of different prebiotic substrates. Panels (**A1**–**A3**) correspond to isolate LBM-Ti173, while panels (**B1**–**B3**) correspond to isolate LBM-Ti195. Panels (**A1**,**B1**) represent bacterial growth in the presence of fructooligosaccharides (FOS), panels (**A2**,**B2**) correspond to mannan-oligosaccharides (MOS), and panels (**A3**,**B3**) represent growth in the presence of inulin.

The growth profiles of *L. reuteri* LBM-Ti173 and *L. reuteri* LBM-Ti195 in media supplemented with different prebiotic substrates are shown in Figure 6. Microbial growth in media containing prebiotics was compared with growth in glucose-free MRS medium. Fructooligosaccharide (FOS) supplementation positively affected the growth of both strains, with 3% (*w*/*v*) FOS resulting in an approximately 2.4-fold increase in growth relative to the control. Inulin supplementation at the highest tested concentration also increased growth, with approximately 2-fold and 3-fold increases observed for LBM-Ti173 and LBM-Ti195, respectively. In contrast, supplementation with mannanoligosaccharide (MOS) did not substantially increase growth compared with the control medium.

Comparison between strains indicated that LBM-Ti195 showed lower growth in the glucose-free control medium than LBM-Ti173, suggesting possible differences in substrate utilization under the tested conditions. However, given the very high genomic similarity between the two isolates, these phenotypic differences should be interpreted cautiously and may reflect discrete genetic variation (e.g., single nuclotide polymorphism), plasmid content, differences in gene regulation, physiological state of the cultures, or experimental variation rather than clearly distinct carbon metabolism strategies.

- In silico comparative analysis of CAZymes in *L. reuteri* type and probiotic strains

**Figure 7:**
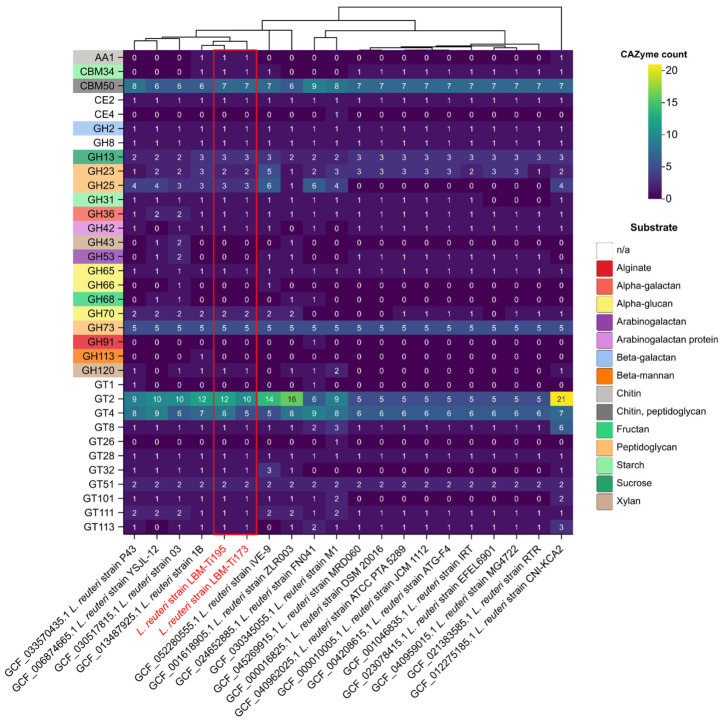
Distribution of predicted CAZyme families in *L. reuteri* LBM-Ti173 and LBM-Ti195, and additional *L. reuteri* genomes selected based on ANI similarity. The profile includes glycoside hydrolase (GH) and glycosyltransferase (GT) families relevant to carbohydrate metabolism and cell-surface glycan-associated functions. The two *L. reuteri* strains sequenced in the present study (LBM-Ti173 and LBM-Ti195) are highlighted in red text and enclosed by a red box for emphasis.

Analysis of CAZyme profiles provided genomic insights into potential carbohydrate utilization capabilities in both *L. reuteri* isolates. LBM-Ti195 and LBM-Ti173 harbored genes assigned to the GH13, GH36, and GH70 families, which are commonly associated with α-glucan/starch metabolism, raffinose-family oligosaccharide metabolism, and sucrose-derived glucan metabolism, respectively (Figure 7). Conversely, the absence of predicted mannan-degrading enzymes, including GH113 family enzymes, in both genomes is consistent with the lack of increased growth on MOS under the tested conditions. Notably, GH113 family genes were also absent from the genomes of the *L. reuteri* type strain DSM 20016 and the probiotic strain *L. reuteri* ATCC PTA 5289.

Compared with these reference strains, LBM-Ti195 and LBM-Ti173 exhibited a higher number of predicted CAZyme terms. Both isolates harbored higher copy numbers of glycosyltransferase families GT2 (12 and 10 copies, respectively) and GT4 (8 and 15 copies, respectively), compared with 6 GT2 and 5 GT4 copies in both *L. reuteri* DSM 20016 and *L. reuteri* ATCC PTA 5289 (Figure 7). These differences suggest variation in cell-surface carbohydrate metabolism and glycan-associated biosynthetic potential, although their functional implications for exopolysaccharide production, adhesion, or gastrointestinal persistence require experimental validation.

## 4. Discussion

### 4.1. Bioprospecting, Identification and Safety

The search for alternatives to antibiotic use in animal production, without compromising productivity and food safety, remains one of the major challenges faced by the scientific community. Probiotics have become established as a recurrent strategy to promote animal health and support zootechnical performance. However, probiotic effects are strain-specific, requiring detailed phenotypic, genomic, and safety assessments for each candidate strain. In the present study, a bioprospecting approach was applied to LAB isolated from the cecal microbiota of broiler chickens, using selection criteria based on: (i) pathogen-inhibitory activity against *S. aureus* [73], a pathogen of veterinary and zoonotic relevance, and (ii) the ability to produce folate (vitamin B9) [74], an essential micronutrient involved in one-carbon metabolism, nucleotide synthesis, and cellular proliferation. In addition, preliminary safety screening considered the absence of hemolytic activity and negative catalase, coagulase, and gelatinase reactions as criteria for selecting isolates for further characterization.

Although MRS [75] medium is commonly used to favor the growth of LAB, the recovery of other Gram-positive microorganisms from cecal samples may still occur, particularly members of the genera *Bacillus* and *Staphylococcus*. Therefore, catalase, coagulase, and gelatinase assays were used as preliminary phenotypic screening tools rather than as definitive taxonomic markers. Catalase negativity is consistent with LAB physiology, whereas coagulase and gelatinase activities are more commonly associated with non-LAB taxa or pathogenic traits in specific bacterial groups. Accordingly, isolates LBM-Ti195 and LBM-Ti173, which showed Gram-positive staining, absence of hemolytic activity, and negative catalase, coagulase, and gelatinase reactions, were selected for genome-based identification and further functional assessment.

The draft genome sizes of LBM-Ti195 and LBM-Ti173 were consistent with the expected genome size range reported for *L. reuteri* and with the compact genome architecture commonly observed in host-associated LAB. Average nucleotide identity (ANI) analysis supported the species-level assignment of both isolates to *L. reuteri*, consistent with the accepted ANI threshold for bacterial species delineation. Comparative genomic analysis against 73 complete RefSeq *L. reuteri* genomes further placed both isolates within the diversity of the species. The high ANI shared between LBM-Ti195 and LBM-Ti173 indicates near-identical genomic backgrounds, although phenotypic differences observed in substrate utilization suggest that discrete genetic, regulatory, plasmid-related, or physiological differences may exist between them.

According to the updated “List of QPS status recommended biological agents” supporting EFSA risk assessments, *L. reuteri* is included among biological agents with Qualified Presumption of Safety (QPS) status [76]. However, QPS status is not unconditional at the strain level. For microbial strains intended for use in food or feed applications, the absence of acquired antimicrobial resistance genes against clinically relevant antimicrobials remains a central safety requirement. This requirement is particularly relevant because acquired AMR determinants may be horizontally transferred to other microorganisms in the gastrointestinal tract, including opportunistic pathogens, thereby contributing to the dissemination of antimicrobial resistance [24].

In this context, the detection of *tetW*, a tetracycline resistance determinant encoding a ribosomal protection protein, represents the major biosafety limitation of LBM-Ti173 and LBM-Ti195. Phenotypic MIC assays showed tetracycline resistance in both strains, and genomic screening identified *tetW* as an acquired AMR determinant. The limited and uneven occurrence of *tetW* among species-level *L. reuteri* RefSeq genomes supports its classification as an acquired rather than intrinsic trait. Therefore, although both isolates showed otherwise favorable safety-associated features, including absence of hemolytic activity and absence of conserved virulence factors under the applied screening criteria, the presence of *tetW* prevents a direct conclusion of suitability for zootechnical application.

The genomic context of *tetW* further reinforces the need for caution. Plasmid-targeted assembly, PLASMe classification, contig coverage, and synteny with plasmid-associated reference regions support a putative plasmid-associated location for *tetW* in both isolates. If confirmed, this would increase the relevance of horizontal transfer risk and would require additional biosafety evaluation. A relevant precedent is the commercial probiotic *L. reuteri* ATCC 55730, which carried resistance determinants on plasmids and was replaced by the plasmid-cured derivative DSM 17938 [77]. This precedent indicates that plasmid curing may represent a feasible future strategy for mitigating this biosafety limitation if *tetW* is confirmed to be located on a non-essential plasmid. However, in the present study, plasmid curing was not performed, transferability was not experimentally tested, and the maintenance of probiotic-associated traits after removal of the *tetW*-bearing element remains unknown. Therefore, any future development of LBM-Ti173 and LBM-Ti195 as zootechnical probiotic candidates must require confirmation of *tetW* removal, restoration of tetracycline susceptibility, genomic stability, and preservation of the functional traits described here.

### 4.2. Probiotic-Associated Traits

Although the selected LAB isolates belong to a bacterial group with a documented history of health-associated applications, they cannot be automatically classified as probiotics. According to current scientific standards, probiotic status requires evidence of a beneficial effect on the host, preferably supported by appropriate in vivo validation [78]. Indeed, several *L. reuteri* strains have been successfully commercialized for years, demonstrating robust safety profiles and diverse host-specific benefits. In human nutrition, strains such as DSM 17938 and NCIMB 30242 [79] are widely utilized to support gastrointestinal and cardiovascular health, having achieved Generally Recognized as Safe (GRAS) status by the US FDA. Similarly, in the veterinary sector, commercial strains like DSM 32203 [80] and DSM 32264 [81] are marketed as zootechnical feed additives, having met the Qualified Presumption of Safety (QPS) criteria established by the EFSA. These veterinary strains have demonstrated significant in vivo efficacy, positively modulating parameters such as body weight, body condition score, fecal characteristics, and microbiota composition in Persian cats [82]. However, existing commercial strains cannot be assumed to meet the distinct physiological and immunological challenges of broiler chickens. This underscores the necessity of isolating novel, chicken-derived strains tailored to this sector. Driven by these industry-specific requirements, the bioprospecting of LBM-Ti173 and LBM-Ti195 was strategically designed to target native avian strains with functional traits, including vitamin synthesis and pathogen-inhibitory activity against poultry pathogens.

#### 4.2.1. Pathogen-Inhibitory Activity

Fermentations mediated by LAB can yield bioactive metabolites with antimicrobial properties against selected bacterial competitors and pathogens. In this study, the cell-free supernatants (CFS) of LBM-Ti173 and LBM-Ti195 showed differential pathogen-inhibitory activity against bacterial indicators of veterinary and food safety relevance. Both isolates exhibited strong inhibition of *L. monocytogenes* CECT 934, suggesting that these strains produce extracellular compounds with activity against this Gram-positive pathogen. Although *L. monocytogenes* is widely recognized for causing severe opportunistic infections in humans [83], it is also relevant in animal production systems, particularly in livestock species such as ruminants [84].

The CFS of LBM-Ti173 and LBM-Ti195 also inhibited *C. jejuni* CCAMP 162, a Gram-negative pathogen of major relevance to poultry production and food safety [85]. In poultry, *C. jejuni* colonization is often asymptomatic but represents an important reservoir for human infection through the food chain [86]. Therefore, the observed in vitro inhibition is relevant as a preliminary indication of antagonistic potential, although it does not demonstrate pathogen control in vivo. Inhibition of *S. aureus* CECT 239 and *C. perfringens* Type A was more moderate, indicating that the antimicrobial spectrum of these CFS preparations is target-dependent.

Given the veterinary and zoonotic relevance of *S. aureus*, a preliminary factorial design was used to evaluate whether culture conditions influenced anti-*S. aureus* activity. Within the tested experimental range, pH 5.0 and 80 rpm favored higher inhibitory activity, with inhibition increasing relative to the control condition. This result suggests that environmental parameters can influence the production or activity of pathogen-inhibitory agents by these isolates. However, these conditions should be interpreted as the most favorable among those tested, rather than as a fully optimized production condition. Moreover, although such findings may inform future production or formulation strategies, they cannot be directly extrapolated to the intestinal environment without additional validation.

Genome mining did not identify classical bacteriocins in LBM-Ti195 and LBM-Ti173. BAGEL4 initially annotated low-confidence Enterolysin_A-like regions, but manual inspection indicated that one predicted region was associated with prophage-related structural genes, including a phage tail protein, and is therefore unlikely to represent a canonical bacteriocin locus. The genomic context of this locus, including additional phage structural components such as head or capsid proteins, supports its interpretation as a prophage-associated region rather than a functional tailocin-like antimicrobial system [87].

In contrast, another predicted region aligned with an M23-family metallopeptidase. Members of this family may participate in peptidoglycan hydrolysis and can be associated with antibacterial activity in some contexts [88,89]. Therefore, the M23-family metallopeptidase identified here represents a plausible candidate associated with the proteinaceous pathogen-inhibitory activity observed in the CFS assays. Nevertheless, its direct role remains to be experimentally demonstrated through approaches such as proteomic detection in the CFS, purification of the active fraction, heterologous expression, or genetic disruption of the candidate locus.

In addition to proteinaceous antimicrobial candidates, antiSMASH predicted a Type III polyketide synthase (T3PKS)-like biosynthetic gene cluster in both strains. The predicted core gene was annotated as containing a chalcone/stilbene synthase-like domain and showed similarity to hydroxymethylglutaryl-CoA synthase after manual curation. Although polyketide-derived metabolites can have antimicrobial properties in other biological systems, the complete loss of inhibitory activity after trypsin treatment indicates that the active antimicrobial component detected under the tested conditions is likely proteinaceous. Thus, the T3PKS-like cluster is unlikely to be the main determinant of the observed CFS-mediated inhibition, although its functional relevance remains unexplored.

#### 4.2.2. Prebiotic Fermentation and CAZyme Profiles

Prebiotics are substrates that can be selectively utilized by host-associated microorganisms and may contribute to host health when incorporated into appropriate formulations [49]. Among them, fructooligosaccharides (FOS) and inulin are widely studied due to their stability and capacity to stimulate selected beneficial bacterial groups [50,51]. In this context, LBM-Ti195 and LBM-Ti173 showed increased growth in vitro in the presence of FOS and inulin, supporting their potential compatibility with formulations containing these prebiotic substrates. Conversely, mannanoligosaccharide (MOS) did not increase growth under the tested conditions, suggesting that these strains may not efficiently utilize MOS as a carbon source in the assay conditions used here.

The CAZyme profiles provided genomic support for some of these phenotypic observations. Both isolates lacked predicted GH113 family enzymes, which are associated with mannan degradation [90], consistent with the absence of increased growth on MOS. Similar absence of GH113 was also observed in the type strain *L. reuteri* DSM 20016 and the probiotic strain *L. reuteri* ATCC PTA 5289. In contrast, both isolates harbored CAZyme families associated with carbohydrate metabolism, including GH13, GH36, and GH70, suggesting potential capacity to metabolize carbohydrate substrates (e.g α-glucan/starch raffinose-family oligosaccharide metabolism, and sucrose-derived glucan metabolism) [91].

Compared with *L. reuteri* DSM 20016 and ATCC PTA 5289, the two avian isolates showed higher predicted copy numbers of glycosyltransferase families GT2 and GT4. These families include enzymes involved in diverse carbohydrate-associated processes, including cell wall biosynthesis and glycan or exopolysaccharide-associated pathways [92]. Therefore, the observed differences may indicate variation in cell-surface carbohydrate metabolism and glycan biosynthetic potential. However, higher GT2 and GT4 copy numbers should not be interpreted as direct evidence of increased EPS production, adhesion, biofilm formation, or gastrointestinal persistence. These functional implications require targeted phenotypic validation, including EPS quantification, aggregation assays, biofilm assays, adhesion assays, and evaluation of survival under gastrointestinal conditions.

Although LBM-Ti195 and LBM-Ti173 share a very high ANI value of 99.99%, the observed differences in growth profiles and CAZyme copy numbers suggest the presence of strain-level phenotypic variation despite their near-identical genomic backgrounds. These differences may reflect subtle genetic, plasmid-related, regulatory, or physiological variation between the isolates. While additional analyses would be required to define the precise molecular basis of these differences, the combined phenotypic and genomic data indicate that both strains retain relevant carbohydrate-associated traits, including the ability to grow in the presence of FOS and inulin, supporting their potential compatibility with selected prebiotic-based formulations. Nevertheless, based on this preliminary screening, LBM-Ti173 appears to present a slight advantage over LBM-Ti195 for future probiotic development.

#### 4.2.3. Folate Biosynthesis and One-Carbon Metabolism as Nutraceutical Determinants

The identification of genes associated with de novo THF synthesis in both strains, encompassing the *folE–folB–folK–folP–folC–folA* pathway, is consistent with the folate biosynthetic capacity previously described in this species [93]. The presence of two *folC*-like paralogs in each genome is also consistent with previous reports in *L. reuteri*, in which distinct FolC-related functions have been associated with folate polyglutamylation and the synthesis of 5,10-CH=THF [19]. These genomic features support the predicted capacity of LBM-Ti173 and LBM-Ti195 to synthesize and process folate derivatives.

The simultaneous identification of genes associated with the “one carbon pool by folate” module (KEGG map00670) further supports a predicted connection between de novo folate biosynthesis and downstream C1 metabolism, including pathways related to purine biosynthesis, thymidylate synthesis, methionine metabolism, and S-adenosylmethionine production [94]. This metabolic potential is relevant in the context of poultry nutrition, since folate participates in fundamental physiological processes linked to cellular proliferation, intestinal epithelial turnover, immune function, and growth [95]. The conservation of the *folE–folB–folK–folC–folP* genomic architecture relative to reference *L. reuteri* strains recognized as folate producers further supports the reliability of the in silico reconstruction, although direct enzymatic activity and in vivo contribution to host folate availability remain to be demonstrated.

The in vitro assessment supported the folate-producing capacity predicted by the genomic reconstruction. Several LAB are known to synthesize folate (vitamin B9), an essential micronutrient for human and animal homeostasis, and this trait is highly strain-dependent [96]. In the present study, both isolates produced detectable folate in FACM, with total concentrations ranging from 42.1 to 50.8 ng/mL. Folate production was relatively similar between LBM-Ti173 and LBM-Ti195, which is consistent with their comparable folate biosynthesis gene profiles.

The folate levels detected in these strains were comparable to, or relatively higher than, values previously reported for selected *L. reuteri* and other probiotic strains cultivated in folate-free media [96,97]. For instance, Jiang et al. [93] reported a maximum extracellular folate production of 60.72 ng/mL among 42 strains isolated from human breast milk, and *folA* knockout increased extracellular folate production approximately three-fold, reaching 150.0 ng/mL in FACM. These comparisons suggest that LBM-Ti173 and LBM-Ti195 belong to the group of *L. reuteri* strains with measurable folate-producing capacity under defined in vitro conditions.

Taken together, the combined genomic and phenotypic evidence indicates that folate production represents one of the most relevant probiotic-associated traits of LBM-Ti173 and LBM-Ti195. In poultry production, folate-producing strains may be of interest because inadequate folate availability can impair physiological functions, growth, and reproductive performance [98]. Folate supplementation has also been associated with improvements in egg-related parameters, including folate concentrations in yolk and albumen, eggshell thickness, and egg mass [99]. Therefore, although the present study does not directly demonstrate improved folate availability or zootechnical performance in birds, these findings support the potential of LBM-Ti173 and LBM-Ti195 as candidates for future in vivo studies evaluating microbial folate production as a functional trait in poultry nutrition.

## 5. Conclusions

In conclusion, this study demonstrates the value of an integrated bioprospecting strategy combining phenotypic screening, genomic characterization, and biosafety assessment to identify avian-derived LAB with probiotic-associated traits. By isolating strains from the cecal microbiota of broiler chickens, the workflow targeted bacteria originating from a relevant gastrointestinal niche for poultry production. The selected *L. reuteri* strains, LBM-Ti173 and LBM-Ti195, displayed several traits of interest, including antimicrobial activity against bacterial indicators of veterinary and food safety relevance, detectable folate production, and growth in the presence of selected prebiotic substrates, particularly FOS and inulin.

These findings support the potential of LBM-Ti173 and LBM-Ti195 as promising candidates for future development of functional zootechnical formulations. However, their transition from probiotic-associated candidates to established probiotics requires additional validation. In particular, in vivo studies are needed to determine whether the observed in vitro traits translate into measurable benefits for poultry health, intestinal function, pathogen control, nutrient availability, or zootechnical performance.

Regarding biosafety, both strains were non-hemolytic and lacked conserved virulence factors under the applied screening criteria. Nevertheless, the detection of phenotypic tetracycline resistance and an acquired *tetW* determinant represents a major limitation for direct application. The putative plasmid-associated context of *tetW* indicates that additional regulatory and experimental evaluation is required, including confirmation of plasmid localization, assessment of transferability, and mitigation of the AMR determinant. If *tetW* is confirmed to be located on a non-essential plasmid, plasmid curing may represent a feasible strategy to generate safer derivative strains, following the precedent of *L. reuteri* DSM 17938. However, such derivatives would require confirmation of *tetW* removal, restoration of tetracycline susceptibility, genomic stability, and preservation of the functional traits described here.

Overall, this work identifies LBM-Ti173 and LBM-Ti195 as biosafety-limited but biologically promising *L. reuteri* candidates, highlighting both their functional potential and the importance of rigorous genomic surveillance in the development of next-generation probiotics under a One Health framework.

## Figures and Tables

**Table 1:** Phenotypic and Safety Characteristics of LBM-Ti173 and LBM-Ti195 strains. Evaluation of general features (Gram, folate/vitamin B9 production, fermentation, gastrointestinal tolerance) and biochemical profiles (coagulase, hemolysis, gelatinase, catalase). Data represent qualitative consensus from independent experimental replicates.

Strain		LBM-Ti173	LBM-Ti195
General features	Gram staining	positive	positive
	Folate (Vitamin B9) production	positive	positive
	Fermentation	hetero	hetero
	Acid pH and physiological bile salt condition	tolerant	tolerant
Virulence test	Coagulase	negative	negative
	Hemolysis	γ	γ
	Gelatinase	negative	negative
	Catalase	negative	negative

**Table 2:** Genomic features of sequenced *L. reuteri* strains (LBM-Ti195 and LBM-Ti173).

Feature	LBM-Ti195	LBM-Ti173
Total reads	5,314,768	5,490,208
Total length (bp)	2,093,541	2,061,835
Number of contigs	75	163
Largest contig (bp)	167,165	63,698
N50 (kb)	19.62	19.62
L50	12	31
L90	42	99
N90 (bp)	15.495	6.676
GC content (%)	38.71	38.84
Coverage	230×	225×
BUSCO completeness (%)	99.8	99.8
CheckM completeness (%)	99.46	99.46
CheckM contamination (%)	0.14	0.14
CDSs	2103	2,085
tRNAs	20	17
rRNAs	2	2
ncRNAs	4	4
tmRNAs	1	1
Pseudogenes	17	17
Coding density (%)	88.9	89.3

**Table 3:** MICs of clinically relevant antibiotics for *L. reuteri* strains LBM-Ti173 and LBM-Ti195. MIC values (expressed in µg/mL) are compared against the EFSA epidemiological cut-off values to determine susceptibility.

Antibiotic/Strain	LBM-Ti173	LBM-Ti195	
	MIC/Result	MIC/Result	Cut-Off Values
Clindamycin	<0.125 (S)	<0.125 (S)	4
Ampicillin	1.0 (S)	1.0 (S)	2
Chloramphenicol	4.0 (S)	4.0 (S)	4
Erythromycin	<0.125 (S)	<0.125 (S)	1
Gentamicin	0.5 (S)	0.5 (S)	8
Kanamycin	16 (S)	16 (S)	64
Streptomycin	8.0 (S)	8.0 (S)	64
Tetracyclin	>64.0 (R)	>64.0 (R)	32
Vancomycin	>64.0	>64.0	NR

Subtitle: NR = Not required; S = susceptible; MR = mostly resistant; R = resistant.

**Table 4:** Extracellular (EC), intracellular (IC) and total folate content (ng mL^−1^) FACM after 24 h at 37 °C.

Antibiotics/Strain	Extracellular (EC)	Intracellular (IC)	Total
*L. reuteri* LBM-Ti173	32.0 ± 3.7	10.2 ± 2.8	42.1 ± 4.6
*L. reuteri* LBM-Ti 195	36.8 ± 5.2	14.1 ±0.5	50.8 ± 4.7

## Data Availability

The original contributions presented in this study are included in the article/Supplementary Materials. Further inquiries can be directed to the corresponding author.

## Supplementary Materials

The following supporting information can be downloaded at: https://www.mdpi.com/article/10.3390/ani16132039/s1, Table S1: Blast results for *tetW* gene conservation within *L. reuteri* genomes; Table S2: Folate metabolic intermediates predicted for each pathway as predicted for strains LBM-Ti173 and LBM-Ti195. Other *L. reuteri* strains are included below as comparative reference; Table S3: Phage genes at intact regions predicted by PHASTEST tool, for strains LBM-Ti173 and LBM-Ti195, respectively.

## Abbreviations

The following abbreviations are used in this manuscript:

AMR	Antimicrobial Resistance
ANI	Average Nucleotide Identity
ANOVA	Analysis of Variance
BGC	Biosynthetic Gene Cluster
BLAST	Basic Local Alignment Search Tool
CARD	Comprehensive Antibiotic Resistance Database
CAZyme	Carbohydrate-Active Enzyme
CDS	Coding DNA Sequence
CFU	Colony-Forming Unit
COG	Clusters of Orthologous Groups
dDDH	Digital DNA-DNA Hybridization
DNA	Deoxyribonucleic Acid
EFSA	European Food Safety Authority
FOS	Fructooligosaccharides
GRAS	Generally Recognized as Safe
HPLC	High-Performance Liquid Chromatography
HMMER	profile Hidden Markov Models
KEGG	Kyoto Encyclopedia of Genes and Genomes
LAB	Lactic Acid Bacteria
LBM	Laboratório de Biotecnologia Microbiana
Mb	Megabase
MIC	Minimum Inhibitory Concentration
MLST	Multi-Locus Sequence Typing
MRS	de Man, Rogosa and Sharpe
NCBI	National Center for Biotechnology Information
ORF	Open Reading Frame
PCR	Polymerase Chain Reaction
PGAAP	Prokaryotic Genome Annotation Pipeline
QPS	Qualified Presumption of Safety
RAST	Rapid Annotations using Subsystems Technology
RGI	Resistance Gene Identifier
SD	Standard Deviation
SEM	Standard Error of the Mean
SNP	Single Nucleotide Polymorphism
TYGS	Type Strain Genome Server
VFDB	Virulence Factor Database
WGS	Whole Genome Sequencing
5,10-CH=THF	5,10-methenyltetrahydrofolate-polyglutamate
